# Ambient air pollution in gastrointestinal endoscopy unit; rationale and design of a prospective study

**DOI:** 10.1097/MD.0000000000013600

**Published:** 2018-12-10

**Authors:** Chang Seok Bang, Keunwook Lee, Jae Ho Choi, Jae Seung Soh, Ji Young Hong, Gwang Ho Baik, Dong Joon Kim

**Affiliations:** a Department of Internal Medicine; b Institute of New Frontier Research; c Department of Biomedical Science, Hallym University College of Medicine, Chuncheon, Korea.

**Keywords:** air pollution, endoscopy, gastrointestinal, indoor, particulate matter, volatile organic compounds

## Abstract

**Background:**

A gastrointestinal endoscopy unit is frequently exposed to gastrointestinal gas expelled from patients and electrocoagulated tissue through carbonation for the treatment of gastrointestinal neoplasms or hemostasis of gastrointestinal bleeding. This can be potentially harmful to the health of not only the healthcare personnel but also patients who undergo endoscopic examinations. However, there has been scarce data on air quality in the endoscopy unit. This study aimed to measure the air quality in the gastrointestinal endoscopy unit.

**Methods:**

This is a prospective study using conventional portable passive air quality monitoring sensors in the gastrointestinal endoscopy unit. We will check the 6 main indoor air quality indices, as well as the atmospheric temperature, pressure, and humidity in the endoscopy unit of a single hospital in Korea. These indices are as follows: carbon dioxide (CO_2_), total volatile organic compounds (VOCs), particulate matter that has a diameter of <2.5 μm, nitrogen dioxide (NO_2)_, carbon monoxide (CO), and ozone. The indices will be checked in the endoscopy unit, including the procedural area, recovery area, and area for disinfection and cleansing of equipment, at 1-minute intervals for at least 1 week, and the type and number of endoscopic procedures will also be recorded. The primary outcome of this study is to determine whether the air quality indices exceed safety thresholds and whether there is any association between ambient air pollution and the type and number of endoscopic procedures.

**Conclusion:**

The results of this study will provide evidence for health-related protective strategies for medical practitioners and patients in the endoscopy unit.

## 1 Introduction

Ambient air pollution is known to be associated with various illnesses and health-related burdens. Causal relationships between particulate matter that has a diameter of <2.5 μm (PM_2.5_) exposure and mortality in chronic obstructive pulmonary disease, lung cancer, or cerebrocardiovascular disease have been elucidated in previous epidemiologic studies.^[1,2]^ Ambient PM_2.5_ was the fifth-ranking mortality risk factor, which accounted for 4.2 million deaths and 103.1 million disability-adjusted life-years (DALYs), and exposure to ozone (O_3_) was the cause of an additional 254000 deaths and the loss of 4.1 million DALYs from chronic obstructive pulmonary disease in 2015.^[3]^ Parenteral total volatile organic compounds (VOCs) exposure during the fetal period was also implicated to adversely influence postnatal growth and neurobehavioral development in the early life stage.^[4,5]^

Indoor air quality is also drawing attention with regard to the increasing level of indoor air pollutants.^[6]^ The level of air pollutants, such as carbon dioxide (CO_2_), VOCs, PM_2.5_, nitrogen dioxide (NO_2_), carbon monoxide (CO), and ozone, emitted from construction materials, furnishings, or consumer chemical products is known to be higher in some indoor environment than those outdoors.^[6,7]^ Chronic exposure to these pollutants is associated with reduced pulmonary function and respiratory symptoms, leading to allergic rhinitis or asthma,^[6,8,9]^ and cardiovascular diseases or cancers, which could be fatal.^[10]^

Healthcare personnel in the endoscopy unit usually spend most of their time in a confined space. A gastrointestinal endoscopy unit is frequently exposed to gastrointestinal gas expelled from patients during endoscopic examination and electrocoagulated tissue through carbonation for the treatment of gastrointestinal neoplasms or hemostasis of gastrointestinal bleeding (through procedures using electrothermal coagulation devices). Moreover, the disinfection and cleansing area for endoscopic equipment is frequently exposed to various chemical detergents and disinfectants. This can be potentially harmful to the health of not only medical practitioners but also patients who undergo endoscopic examinations. Moreover, ambient air pollution in the endoscopy unit can potentiate endoscopy-related infections. However, there has been scarce data on air quality measurement in the endoscopic unit.^[11,12]^ The aim of this study is to check the air quality in the gastrointestinal endoscopy unit.

## 2 Methods

### 2.1 Overview of the study design

This is a prospective study using conventional portable passive air quality monitoring sensors in the gastrointestinal endoscopy unit. The uHoo filter (©uHoo Limited, Hong Kong) is a validated passive air sampling device for conventional indoor air toxin monitoring. The 9 air quality sensors use safety thresholds indicated by the United States Environmental Protection Agency (US EPA) and the World Health Organization (WHO). The sensors in the uHoo filter uses traceability standards from various bodies, such as Underwriters Laboratories Inc (UL) and the National Institute for Occupational Safety and Health (NIOSH) and utilized controlled chambers in laboratories to validate the tolerance ranges of each sensor.

We will check the 6 main indoor air quality indices with atmospheric temperature, pressure, and humidity in the endoscopy unit of Hallym University Chuncheon Sacred Heart Hospital in Korea. These indices are as follows: CO_2_, VOCs, PM2.5, NO_2_, CO, and ozone. The indices will be checked in the endoscopy unit, including the procedural area, recovery area, and area for disinfection and cleansing of equipment, at 1-min intervals for at least 1 week, and the type and number of endoscopic procedures will be recorded. The recording period is from June 2018 to November 2019. Data will be collected from each area of the endoscopy unit and restored into a mobile application, and the restored data will be transferred into the statistical software and analyzed after the recording period.

### 2.2 Study subjects and interpretation standards of air quality indices

This is not a human subject study, and only the air quality indices in the endoscopy unit, including the procedural area, recovery area, and area for the disinfection and cleansing of equipment and the type and number of endoscopic procedures are the variables of study. There are no current standards for indoor air quality in the endoscopy unit. Therefore, thresholds of the manufacturer's protocol according to the United States Occupational Safety and Health Administration, the US EPA, and the WHO have been adopted in this study.

The CO_2_ level between 400 and 800  parts-per-million (ppm) will be considered as acceptable indoor air quality in the endoscopy unit. A level between 800 and 1500 ppm will be considered as the tolerable state, but external air needs to flow and CO_2_ needs to be emitted by ventilation. A level >1500 ppm will be considered as poor indoor air quality, which will be likely reached only in unusual circumstances.

A total VOC concentration between 0 and 400 parts-per-billion (ppb) will be considered as acceptable indoor air quality in the endoscopy unit. A concentration between 400 and 800 ppb will be considered as the tolerable state, but removal or reduction of the source of VOCs is needed. A concentration >800 ppb will be considered as poor indoor air quality, which should be avoided.

A PM_2.5_ level <50 μg/m^3^ will be considered as acceptable indoor air quality in the endoscopy unit. A level between 50 and 100 μg/m^3^ will be considered as the tolerable state but might affect patients with underlying respiratory conditions, and reduction of the level is needed. A level >100 μg/m^3^ will be considered as poor indoor air quality, which should be avoided.

An NO_2_ level <100 ppb will be considered as acceptable indoor air quality in the endoscopy unit. A level between 100 and 250 ppb will be considered as the tolerable state, but external air needs to flow and NO_2_ needs to be emitted by ventilation. A level >250 ppb will be considered as poor indoor air quality, which should be avoided.

A CO level <35 ppm will be considered as acceptable indoor air quality in the endoscopy unit. A level between 35 and 70 ppm will be considered as the tolerable state, but external air needs to flow and CO needs to be emitted by ventilation. A level >70 ppm will be considered as poor indoor air quality, which should be avoided.

An ozone level <30 ppb will be considered as acceptable indoor air quality in the endoscopy unit. A level between 30 and 70 ppb will be considered as tolerable state but might affect sensitive individuals. A level >70 ppb will be considered as poor indoor air quality, which can result in respiratory damage and illness.

The atmospheric temperature, pressure, and humidity in the endoscopy unit will be coincidently measured to interpret the results of air quality indices. A level of atmospheric temperature between 21 and 26°C will be considered as acceptable indoor range, and a level between 27 and 40°C will be considered as high temperature, which is conducive to bacterial or fungal growth. An atmospheric pressure between 970 and 1030 millibar (mbar) will be considered as acceptable indoor range, and a level <970 or >1030 mbar will be considered as tolerable but indicating weather change. A level of relative humidity between 30 and 50% will be considered as acceptable indoor range, and a level <30% or between 50 and 100% will be considered as tolerable but indicating too dry or too humid condition. The reference range of indoor air quality indices of this study is presented in Table 1.

**Table 1:**
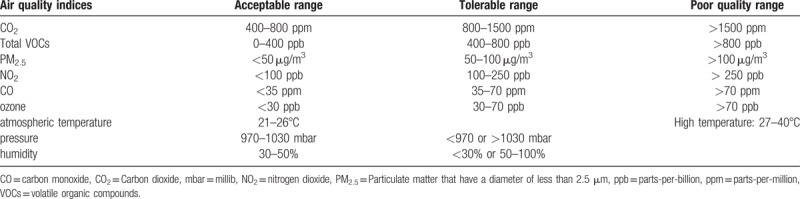
Reference range of air quality indices used in this study.

### 2.3 Outcome measurement

The primary outcome of this study is to determine whether the air quality indices exceed safety thresholds and whether there is any association between ambient air pollution and the type and number of endoscopic procedures. The portable passive air quality monitoring sensors will be installed at endoscopists’ and assistants’ head level in the procedural area and area for disinfection and cleansing of equipment and at patients’ head level in the recovery area to reflect the real breathing air. The air quality indices will be measured in 1-min intervals for at least 1 week, and the type and number of endoscopic procedures will be recorded according to the procedural area, recovery area, and area for disinfection and cleansing of equipment.

### 2.4 Statistical analysis

The sample size was not calculated because there has been no previous study on this topic as well as on the descriptive characteristics of occupational and environmental medicine with regard to this type of study.

Continuous variables will be expressed as mean ± standard deviation or median with interquartile range. Categorical variables will be expressed as number and percentage. First, we will describe the average values of the air quality indices of the endoscopy unit, and the number and type of endoscopic procedures with or without the use of electrocoagulation devices will be also presented in a descriptive manner. Second, we will describe how often tolerable or unacceptable air quality indices were present during the study period. Third, we will perform a Cox proportional hazards regression or logistic regression analysis to identify independent risk factors associated with the level of tolerable or unacceptable air quality indices (whether there is any association between ambient air pollution and the type and number of endoscopic procedures). A *P* value <.05 (2-tailed) will be adopted as the threshold for statistical significance for all tests. All analyses will be performed using SPSS version 24.0. (SPSS Inc., Chicago, IL).

### 2.5 Data management

A common case report form (CRF) was produced by investigators and approved by the Institutional Review Board (IRB) of Hallym University Chuncheon Sacred Heart Hospital. Only designated members of the study staff will be allowed to record and correct data in the CRFs. No personal identifiable variable will be recorded in this study, and the common data form will be produced by the principal investigator at the end of data measurement. Confidential data can be released and provided for the purpose of the safety issue.

### 2.6 Ethics

The study protocol adheres to and will be conducted in accordance with the ethical guidelines established by the 1975 Declaration of Helsinki and the Food and Drug Administration regulations on good clinical practice and follows the Standard Protocol Items: Recommendations for Interventional Trials (SPIRIT).^[13]^ We had received an approval from the IRB of Hallym University Chuncheon Sacred Heart Hospital (number, NON2018-002) before the study was initiated. This study was registered at ClinicalTrial.gov on October 2018 (clinical trial registration number, NCT03724565). Informed consent to participate in the study was exempted by the IRB due to the environmental medicine format of this study and the air quality measurements not relevant to human data or intervention. Healthcare personnel, except for investigators of this study, who routinely perform medical practice in the endoscopy unit, were not informed about the air quality measurement in order to not allow intentional ventilation of endoscopy suites.

## 3 Discussion

Air pollution is defined as the contamination of the indoor or outdoor environment by mixture of any chemical, physical, or biological agent that modifies the natural atmosphere.^[14,15]^ The majority of these components includes ozone, CO_2_, VOCs, PM_2.5_, and NO_2_.^[14,15]^ Causal relationships between ambient air pollution exposure and various conditions, including cardiovascular, respiratory, and neurological diseases or even cancer have been well documented.^[3,10,14,16]^ Linear physiological changes in the cardiovascular and autonomic nervous systems on exposure to CO_2_ at concentrations ranging from 500 to 5000 ppm have been elucidated.^[17]^ VOCs are carbon-based chemicals that easily evaporate at room temperature, and exposure to VOCs is associated with oxidative stress, decreased pulmonary function, and airway inflammation.^[18]^ PM_2.5_ is known to penetrate deep into the alveolar space of the lung, impairing pulmonary function, and even into the bloodstream, leading to cardiovascular diseases or even acute cardiovascular mortality and disability.^[14–16]^ Diesel exhaust PM is also classified as a carcinogen.^[19]^ NO_2_ exposure is associated with decreased pulmonary function and causes bronchitis or bronchopneumonia.^[15,20]^ Attachment of CO to hemoglobin, preventing the transport of adequate oxygen and impairing release of oxygen from the remaining oxyhemoglobin, is presumed to be a major mechanism in CO-related health damage, and a high concentration of CO is associated with heart attack.^[15,20]^ Ozone is formed by atmospheric chemical reactions involving nitrogen oxides and VOC precursor gas emissions in the presence of sunlight.^[21]^ It is associated with the stimulation of transcription factors and the increased expression of cytokine and adhesion molecules, which lead to the development of cardiovascular and respiratory diseases.^[15]^

Indoor air pollution is gaining attention and identifying the source of indoor air pollutants and removing or substituting furnishings and building materials have been recommended to control indoor air quality.^[6,10]^ However, indoor air pollutants in the gastrointestinal endoscopy unit might be different from those in conventional buildings, because there is a possibility that patients-derived gas or endoscopic procedures (especially procedures using electrocoagulation devices) might be the source of indoor air pollution.

Distension of the intestinal lumen is essential to ensure adequate visibility and secure the space required for a safe procedure.^[22]^ A large amount of gas from the endoscopic device is insufflated during procedures.^[22]^ Room air is the most commonly used material during diagnostic or therapeutic endoscopic procedures; however, expelled gas from patients during or after the procedure is presumed to be a mixture of insufflated gas by endoscopic devices and patients’ gastrointestinal gas containing numerous microorganisms. Moreover, therapeutic endoscopic procedures including endoscopic mucosal resection or endoscopic submucosal dissection of gastrointestinal neoplasm using electrocoagulation devices, which could lead to development of carbonated gas in the tissue, have been increasingly performed with the recent advancement in endoscopic skills and expertise. Electrocoagulation devices are also used in emergency procedures, such as hemostasis of gastrointestinal bleeding, and the frequency and exposure to air pollutants from these emergent situations are unpredictable to the healthcare personnel in the gastrointestinal endoscopy unit. Considering that endoscopy personnel usually spend most of their time in a confined space in the endoscopy unit and the patients who undergo endoscopic examinations are also exposed to ambient air in the same area, particular attention is needed to monitor the air quality in the endoscopy unit.

Minimizing the risk of infection in endoscopic procedures is important, and appropriate reprocessing of flexible endoscopes and endoscopic accessories has been emphasized for the safety of patients and quality assurance in gastrointestinal endoscopy.^[23–25]^ Considering that air is a well-recognized medium and inhalation of aerosolized microorganisms is one of the modes of microorganism transmission, studies on ambient air pollution in the endoscopy unit are important.^[25,26]^ The number of microorganisms present in the air of some places also depends on the air quality that is supplied, number of people present, and types of procedures performed in the area.^[26]^ In terms of the health and safety of the endoscopy personnel, universal precautions with wearing appropriate protective clothing with face masks and eyeglasses in people who are involved in the reprocessing procedure are recommended.^[24]^ However, there have been scarce data on air quality measurement in the endoscopy unit and no consideration of ambient air pollution that can potentiate endoscopy-related infections.^[11,12]^ Only specific respiratory diseases that can be spread via an airborne route (e.g., tuberculosis) are documented in the guidelines and special precautions (performing endoscopy in a negative-pressure room), and the use of personal respiratory protection for healthcare personnel who lack immunity to airborne viral diseases has been recommended.^[25]^

This study will continuously monitor the indoor air quality in the endoscopy unit. The strength of this study resides in providing evidence regarding health-related protective strategy of the medical practitioner and patients in the endoscopy unit.

## Author contributions

**Conceptualization:** Chang Seok Bang.

**Data curation:** Chang Seok Bang, Keunwook Lee, Jae Ho Choi, Jae Seung Soh, Ji Young Hong, Gwang Ho Baik, Dong Joon Kim.

**Formal analysis:** Chang Seok Bang, Keunwook Lee.

**Funding acquisition:** Chang Seok Bang, Keunwook Lee.

**Investigation:** Chang Seok Bang, Keunwook Lee, Jae Ho Choi, Jae Seung Soh, Ji Young Hong, Gwang Ho Baik, Dong Joon Kim.

**Methodology:** Chang Seok Bang.

**Project administration:** Chang Seok Bang.

**Resources:** Chang Seok Bang.

**Visualization:** Chang Seok Bang, Keunwook Lee.

**Writing – original draft:** Chang Seok Bang, Keunwook Lee.

**Writing – review & editing:** Chang Seok Bang.

Chang Seok Bang orcid: 0000-0003-4908-5431.
